# UKCAT and medical student selection in the UK – what has changed since 2006?

**DOI:** 10.1186/s12909-020-02214-1

**Published:** 2020-09-05

**Authors:** Rachel Greatrix, Jonathan Dowell

**Affiliations:** 1 University of Nottingham, Nottingham, UK; 2 University of Dundee, Dundee, UK

**Keywords:** UKCAT, Medical student selection, Medical student admissions

## Abstract

**Background:**

The United Kingdom Clinical Aptitude Test (UKCAT) is an aptitude test used since 2006 within selection processes of a consortium of UK medical and dental schools.

Since 2006, student numbers have increased in medical training and schools now have an increased focus on widening access. A growing evidence base has emerged around medical student selection (Patterson et al., Med Educ 50:36–60, 2016) leading to changes in practice. However, whilst some papers describe local selection processes, there has been no overview of trends in selection processes over time across Universities.

This study reports on how the use of the UKCAT in medical student selection has changed and comments on other changes in selection processes.

**Methods:**

Telephone interviews were conducted annually with UKCAT Consortium medical schools. Use of the UKCAT was categorised and data analysed to identify trends over time.

**Results:**

The number of schools using the UKCAT to select applicants for interview has risen, with cognitive test results contributing significantly to outcomes at this stage at many universities. Where schools use different weighted criteria (Factor Method), the UKCAT has largely replaced the use of personal statements. Use of the test at offer stage has also increased; the most significant use being to discriminate between applicants at a decision borderline. A growing number of schools are using the UKCAT Situational Judgement Test (SJT) in selection.

In 2018, all but seven (out of 26) schools made some adjustment to selection processes for widening access applicants. Multiple Mini Interviews (MMIs) are now used by the majority of schools.

Whilst medical student numbers have increased over this time, the ratio of applicants to places has fallen. The probability of applicants being invited to interview or receiving an offer has increased.

**Conclusions:**

More medical schools are using the UKCAT in undergraduate selection processes in an increasing number of ways and with increasing weight compared with 2007. It has replaced the use of personal statements in all but a few Consortium medical schools.

An increased focus on academic attainment and the UKCAT across medical schools may be leading to the need for schools to interview and make offers to more applicants.

## Background

Medical student selection remains a challenging and contentious issue internationally. A review of the Ottawa consensus statement on selection and recruitment to the healthcare professions reported a growing evidence based approach to selection in the UK [1]. However, it also highlighted the continuing complexity of, and challenge, in medical student selection, noting the often conflicting drives around diversity, differential attainment, retention and institutional aspirations.

Traditionally there were three stages in the selection of students for UK medical schools: an initial assessment of academic qualifications alongside a further assessment of qualities obtained from the Universities and Colleges Admissions Service (UCAS) application form (personal statements and references). The outcome of this stage would usually identify those applicants to be invited to interview. Whilst a small number of Universities do not interview, the vast majority of medical schools make offer decisions based on interview outcomes [2]. Selection processes are at the discretion of individual Universities; whilst the core approach to selection may be largely similar, differences exist between schools [3].

The United Kingdom Clinical Aptitude Test (UKCAT) (www.ucat.ac.uk) was created in 2005, growing out of a collaboration between 23 medical schools and eight dental schools. Since then a number of Universities joined and left the Consortium; 30 medical schools used the UKCAT as part of their selection processes in 2019. This included new medical schools created following the expansion of medicine student numbers [4].

The UKCAT Consortium set out to provide medical schools with an additional selection tool, to assist in the challenge of discriminating between the large number of academically high achieving applicants. At the same time Universities were looking for measures (over and above academic achievement and cognitive ability) to identify the traits necessary in applicants to make them good doctors and dentists. From the outset, the Consortium was interested in the extent to which the test predicted performance in medical and dental schools.

The UKCAT originally comprised four cognitive subtests (verbal reasoning, quantitative reasoning, abstract reasoning and decision analysis), providing four subtest scores (each with a scale score range of 300–900) which when totalled produced an overall score (range 1200–3600) for each candidate. The UKCAT Situational Judgement Test (SJT) (targets the non-academic attributes of integrity, perspective taking, team involvement, resilience and adaptability) was introduced in 2013 with candidates allocated to one of four bands with Band 1 being the highest performing candidates. The Decision Analysis subtest was replaced by Decision Making in 2017. In 2019, following international expansion, UKCAT changed its name to University Clinical Aptitude Test (UCAT). Further information regarding UKCAT test content can be found on the website (www.ucat.ac.uk) and in annually produced technical reports e.g. [5].

UKCAT consortium members are informed about content, scoring and statistical performance of the test to enable participating schools to decide how best to use the test e.g. [5]. UKCAT test scores provide standardised measures that schools may use in selection but there is no explicit policy or recommendation made to schools.

Changes and trends in UKCAT’s contribution to medical student selection between 2007 and 2018 (inclusive) are described in this paper. Information was obtained from admission tutors/officers during an annual telephone interview. Data obtained through these interviews represents a unique source of information regarding selection processes for medical programmes more broadly.

The purpose of this paper is to provide information as to how selection to medical schools in the UK has changed and the impact UKCAT has had on these changes. By understanding the changes that occurred over this period, we might better seek to understand the impact of changing selection methods and widening access initiatives.

## Methods

Building upon a previous paper [6], telephone interviews (*n* = 23–26) were undertaken with UKCAT Consortium medical schools on an annual basis. Interviews used a standard questionnaire (Supplementary Document 1) and were conducted each summer term. The interviewees were either admission tutors or administrators familiar with local selection processes. Interviews focussed on selection to the core programme for each school, which in most cases was a five-year undergraduate programme, although the study also includes three schools that only delivered a graduate-entry programme. Results are not reported here for how schools used the test for other than their core programme. Other programmes may include gateway, accelerated and graduate entry programmes. Key points of each interview were noted and a summary sent to each interviewee giving them an opportunity to make corrections. In most cases interviewees confirmed the document as a correct summary of the interview. On occasions, interviewees made minor changes to the document.

### Categorisation of use of the UKCAT in selection

Use of the UKCAT in selection has previously been categorised as Borderline, Factor, Threshold and Rescue Methods [6]. This categorisation of use of the test has been utilised subsequently by others [7, 8] to describe trends in use of the UKCAT over time.

The following example describes how the Uses of the UKCAT in selection might have changed over time for a hypothetical University.

**Table:** 

***University X – Selection Processes for Main Medical Programme*** *In 2006, University X initially rejected those applicants not meeting a minimum academic threshold. The remaining applicants had their academic record (achieved and predicted) and their UCAS Form (personal statement and reference) scored. The scores were combined, contributing 50% each to a total first stage score. Applicants with the highest scores were invited to a traditional interview. Interviewees were then ranked and applicants with the highest scores made offers.*	
*The UKCAT was used for the first time in selection in 2007 and only to discriminate between applicants at a border line (for offer) after interview (****borderline use****), affecting decisions for four applicants.*	
*This use of the UKCAT continued until 2011 when UKCAT was used as an additional criterion in selecting applicants for interview. Academic record contributed 50% towards the first stage score and UKCAT and the UCAS Form both contributed 25% (****factor use, 25% weighting****). The UKCAT continued to be used for borderline applicants after interview.*	
*In 2016, the University ceased scoring the UCAS Form. The first stage score now comprised 60% academic record and 40% UKCAT (****factor use, 40% weighting****). In addition to the academic screen, applicants who had achieved Band 4 in the UKCAT Situational Judgement Test (SJT) were rejected without further consideration (****Threshold Method****).*	
*In the same year the University moved from traditional to multiple mini interviews (MMIs) and discontinued using the Borderline Method.*	

### Data extraction

Data from each questionnaire was extracted into an excel spreadsheet. There were some minor variations (e.g. some Universities did not interview) but selection processes were usually split across three stages: pre-screening, selection for interview, selection for offer.

## Results

The following figures show trends in the use of the UKCAT since the first year of testing. The number of schools using the test has changed over this period with some schools having left the Consortium and others joining.

### Invitation for interview

**Fig. 1:**
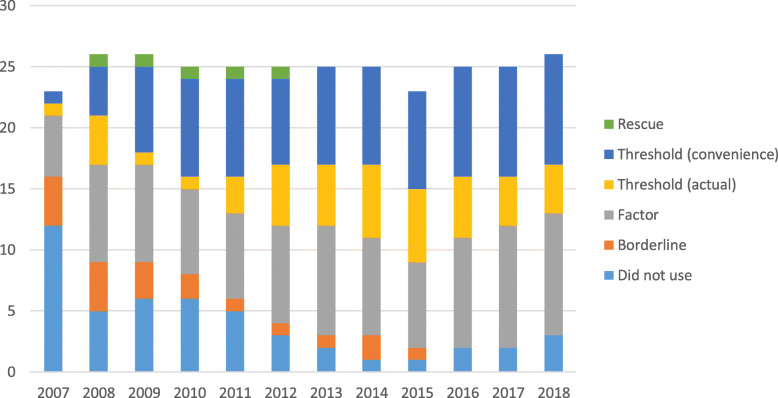
High level summary, use of the UKCAT: Invitation to interview. Use of the UKCAT in selection for interview is as categorised in Table 1

Figure 1 provides a high level summary of test use to select for interview. Use of the test is as categorised in Table 1. In the first year of testing the majority of schools either made no use of the test at this stage (*n* = 12) or only used the test to discriminate between borderline applicants (*n* = 4). Five schools used the Factor Method. By 2018, only three schools were not using the test at this stage; 10 utilised the Factor Method; five applied an actual threshold and eight a convenience threshold. The growth in use over time has been more noted in the Threshold Method. For 2018 entry no schools used the Borderline Method at this stage in their processes.

**Table 1:** Categorising Use of the UKCAT in Selection

Method	Description
**Borderline Method**	UKCAT was used as an objective measure to discriminate between applicants lying at a decision borderline for either interview or offer.
	Some schools included this method in their selection ‘toolkit’ but did not always use it depending on outcomes at other selection stages.
	This method was generally only applied to a small number of applicants and as such we define this use as ‘light touch’.
**Factor Method**	Schools used weighted criteria to create a unique algorithm determining a score for applicants which could then be compared. The Factor Method was used most frequently to determine invite for interview and, on occasion, to make offers.
	Weighted criteria used to identify applicants for interview included academic scoring, UKCAT scoring (usually the UKCAT total score), personal statement scoring and University own questionnaires. Following interview, some Universities weighted the interview score alongside academic, personal statement and other scores.
	An example of how the Factor Method is used in selection is provided below.
	Weighting and the range of scores for different criteria determined impact on outcomes. If, for example, academic score range was limited, then regardless of how low the UKCAT weighting, UKCAT may still have significant impact on outcomes.
**Threshold Method**	Applicants were required to achieve a minimum UKCAT score to progress to the next stage of a selection process. Thresholds were most commonly used to identify those to invite for interview, often applied following an assessment of academic qualifications and/or other criteria.
	‘Actual’ thresholds were pre-determined and often published for the information of applicants. Actual thresholds may have been used to reduce the number of applicants for consideration at a further stage (e.g. to reduce the number of UCAS forms for scoring).
	‘Convenience’ thresholds ranked applicants by UKCAT, choosing the cut off score which provided the N applicants required for interview. Some applicants were not clear as to whether their score would meet this requirement although schools have on occasion published indicative scores to guide applicant choice.
	This method has been regarded as giving UKCAT a higher impact on outcomes than other measures. In some cases however, where cut off scores were low, the impact was less significant, screening out small numbers of applicants.
**Rescue Method**	High UKCAT scores used to ‘compensate’ for a lower score in another part of the selection process, ‘rescuing’ applicants who might otherwise have been rejected. Overall impact of this use was light touch, affecting small numbers of applicants.

The most notable trend has been the decline in the number of schools not using the test or only using the test for borderline applicants; at the same time there has been an increase in schools (2008, *n* = 7; 2018 *n* = 13) applying a threshold.

The introduction of the UKCAT SJT in 2013 gave schools a further criterion for use in selection. Most schools using the SJT at this stage excluded the lowest performers in this subtest (i.e. the 10% candidates (approx.) that achieved a Band 4).

Some schools used the test in more than one way at this stage and this data is provided in Supplementary Document 2.

### Threshold method

**Fig. 2:**
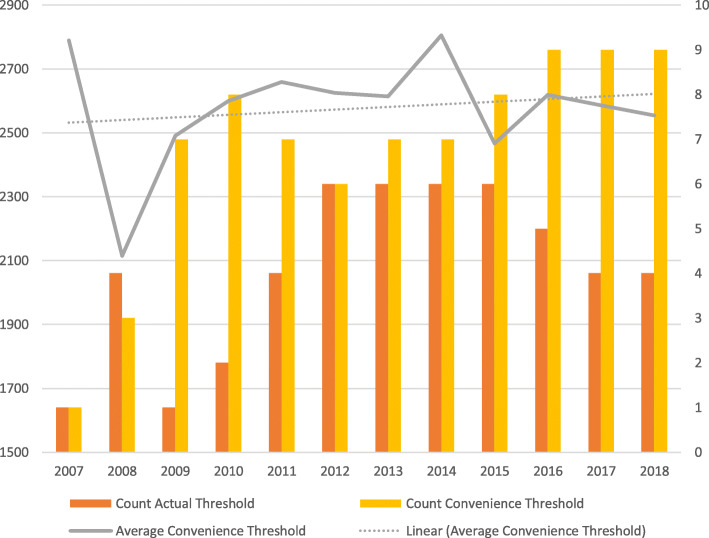
Threshold Method (invitation for interview)

Figure 2 illustrates how the use of threshold scores has changed over time. Additional data recording mean thresholds (actual and convenience) is provided in Supplementary Document 3.

Four schools used the test to pre-screen applicants using a pre-defined ‘actual threshold’ score in 2018. Whilst this is higher than in 2007 (*n* = 1), the maximum number of schools using this method was six (2012–2015). The small increase in average actual threshold scores used by schools in this manner can largely be accounted for by a rise in mean average total scores over this period. Most schools using the test in this way screened out lower performing applicants although there were schools which applied high actual threshold score in some years (2700+).

In 2018, nine schools used a ‘convenience threshold’ UKCAT score to select applicants for interview. This number rose steadily over time from one school in 2007. The average threshold has also increased over time. In 2018 this figure (2544) was only slightly higher than the mean average UKCAT score (2540); in previous years (with one exception) the convenience average score was higher than the overall mean average.

Convenience thresholds applied by individual schools varied over time because decisions made each year by schools relate to each University’s ‘gathered field’. That is, the number of applicants a University may want to interview depends on total application numbers and the quality of those applicants. This is further informed by previous experiences in relation to how many interviews need to be undertaken to arrive at the correct number of offers. This resulted in some schools applying relatively high thresholds at this stage (e.g. three schools had a threshold score 2640+).

### Factor method

**Fig. 3:**
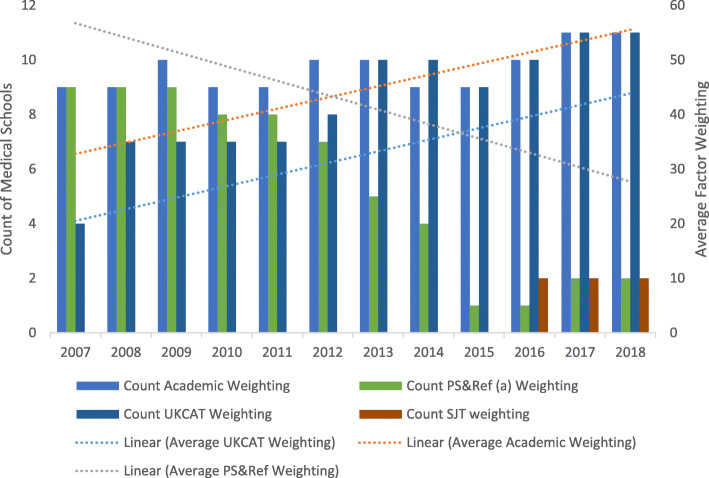
Factor Method (invitation for interview). (a) Personal statement and reference. Use of the UKCAT in selection for interview is as categorised in Table 1

Figure 3 illustrates the number of schools using the Factor Method to identify applicants for interview and the average percentage weighting applied for each criterion in each year. Additional data recording mean average criteria weightings is provided in Supplementary Document 3.

There has been significant change in selection of applicants for interview. The use of academic scores has increased marginally. However, in 2007, nine schools used the personal statement and reference as criteria in selection for interview. By 2018, this number declined to only two schools. In 2007, the personal statement and reference score accounted for (on average) 58% of the weighting for selection for interview and was the highest % weighting used until 2012. By 2018, this figure had declined to 36%. From 2013, the highest % weighting was for academic scoring. In 2007, seven schools used the UKCAT as a criterion at this stage; in 2018 this increased to 11. During this same period, the weighting applied to the UKCAT increased on average from 26 to 39%.

There was an upward trend in the average use of the UKCAT as a criterion for selecting applicants for interview. By 2018, a number of schools applied a high weighting to the UKCAT, with one school using the test as 66% of the weighting to determine whether to invite applicants to interview.

Of the 11 Universities using the UKCAT in this way, there has been a clear increase in weighting for the majority (*n* = 7). For most of these Universities this step change occurs at the point where reliance on the use of personal statements/references reduces or disappears. In four cases the weighting for the UKCAT has remained steady or reduced over time.

In recent years a small number of schools (*n* = 2, 2018), used the SJT subtest as a weighted criterion at this stage in selection.

### Making an offer (Fig. 4)

In the first year of testing, 11 schools used the test to discriminate between borderline applicants at this stage. A further two schools used the Factor Method at this stage. One school applied a UKCAT threshold at offer stage.

**Fig. 4:**
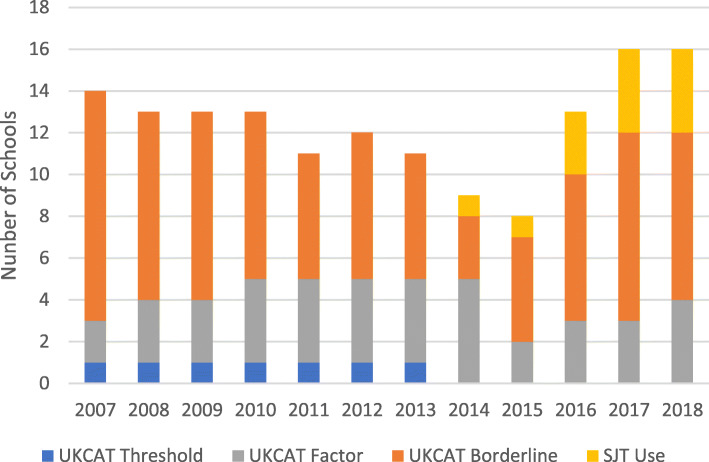
Use of the UKCAT: making an offer. Use of the UKCAT in selection is as categorised in Table 1

By 2018, use of the UKCAT at offer stage remained relatively limited; eight schools used the UKCAT within a Factor Method (four schools using the cognitive test total score and four schools using the SJT). A further eight schools used the test to select between applicants at a borderline after interview.

There has been some recent growth in use of the test at the offer stage with schools identifying this as an appropriate place to include the SJT within selection. In addition to the four schools using the SJT as a weighted criterion (alongside for example interview outcome), a further two schools used the SJT to discriminate between borderline applicants. One school used SJT Band 4 as a potential red flag in MMIs and one applied an SJT threshold for offer.

### Factor method (making an offer)

**Fig. 5:**
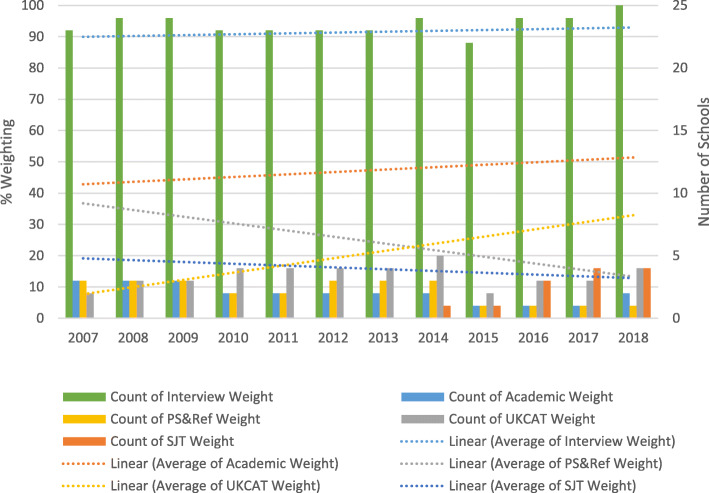
Factor Method (making an offer)

Figure 5 illustrates the extent to which the interview continues to dominate decision making regarding offers to applicants.

In 2018, only four schools used the UKCAT cognitive test scores as a criterion for making an offer to applicants and this number has changed little since 2007. The average weighting of the UKCAT cognitive tests during this period increased from 8 to 27%. Only one school has used the personal statement and reference at this stage since 2015.

In 2018, three schools used the UKCAT SJT as a criterion at this stage.

### Borderline method (Fig. 6)

In 2018 nine schools used the UKCAT to discriminate between applicants at a borderline often in conjunction with other uses. In UKCAT’s early years there was greater use of the test in this way, reflecting a more cautious approach. Two of the schools using the test for borderline applicants used the SJT to discriminate at a borderline for offer.

**Fig. 6:**
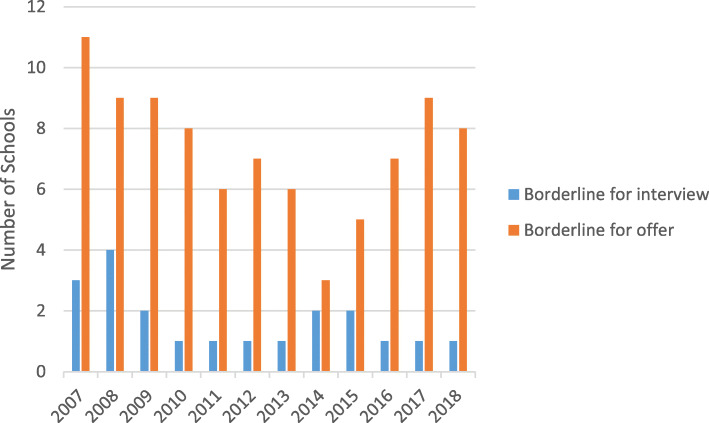
Borderline Method Use of the UKCAT

### Rescue method

It is only possible to identify two schools definitively using a Rescue Method in the early years of UKCAT. One school (in 1 year) invited applicants to interview on the basis that a high UKCAT score might mitigate for a low scored personal statement and reference.

The Rescue Method use, depending on how and when it is applied feels very similar to both the Factor Method (if weighted) or Borderline Method (if introduced after the majority of applicants had been identified for interview).

### Use of the situational judgment test in selection

The UKCAT SJT was introduced operationally in 2013 testing. Schools were cautious to use the test in selection. In 2018 four schools excluded applicants who had achieved a Band 4 SJT. Two schools applied a weighting to the SJT when selecting applicants for interview. Eight schools used the SJT at the offer stage, four as a weighted criterion (some within an MMI), two for borderline applicants, one as a threshold and one as a marker of concern within their MMI processes.

### Multiple use of UKCAT scores

UKCAT scores were used by some schools in more than one way within and at different stages in selection processes. Table 2 summarises use of the UKCAT observed in the 2018 admissions cycle (note number of medical schools = 26).

**Table 2:** Use of the UKCAT by Universities, 2018

Use of the UKCAT (to select for interview)	Number of Schools	Use of the UKCAT (at offer stage)	Number of Schools
**Cognitive Subtests Weighted (Factor)**	12	Cognitive Subtests Borderline	6
**Cognitive Subtests Threshold (Convenience)**	9	Cognitive Subtests Factor	5
**Cognitive Subtests Threshold (Actual)**	5	SJT Weighted (Factor)	4
**SJT Threshold**	4	SJT Borderline	2
**SJT Weighted (Factor)**	2	Other	2
**Cognitive Subtests Borderline**	1		

In 2018, eight Universities used the test at only one point in their selection processes; 12 Universities used the test in two different ways; four Universities used the test in three different ways and two universities used the test at four points in their selection process. The most common ‘secondary’ use of the UKCAT was to distinguish between applicants at borderlines for offer (*n* = 8) and the application of an additional SJT threshold to select for interview (*n* = 4).

### Other findings

#### Widening access

All Universities in the UK are expected to have in place strategies to widen access to higher education by increasing participation of students from under-represented groups with a particular focus on those from lower socio-economic backgrounds. Some Medical Schools offer programmes specifically aimed at these applicants. Universities will also flag (based on demographic data provided) widening access applicants at the point of application, allowing those involved in selection to consider this group separately.

Information was not routinely collected in detail from schools regarding use of the test for widening access programmes and/or applicants. However, respondents were asked to comment on the extent to which use of the test varied between applicant groups. At the same time, some schools provided additional information regarding programmes other than their main programme.

In the first few years of testing very little information was reported by schools regarding adjustments to processes for widening access applicants. In 2008, information was only reported by five schools. In 2018, all but seven schools referred to a specific aspect of their selection processes which had been adjusted for widening access applicants. There were distinctions made in some schools between applicants coming through recognised widening access programmes and other widening access applicants. The detail as to how these two different applicant cohorts were treated was not always provided in sufficient detail for distinctions to be made here.

Some schools did not require some widening access applicants (from access programmes) to take the UKCAT or did not use the test for widening access applicants (flagged at application) (2018, *n* = 5). 12 schools adjusted criterion scoring for widening access applicants. Of these, eight adjusted academic scores and six adjusted UKCAT scores. In most cases this involved adjusting an academic and/or UKCAT score for identified widening access applicants to increase their chances of being invited to interview.

#### Types of interview

In 2007 only two (out of 25) Universities used MMIs as the final stage of their admission processes. By 2018, 21 (out of 26) Universities used MMIs. The significant shift to the use of MMIs took place from 2013 onwards.

#### Applicant numbers

When completing questionnaires, admission tutors provided information regarding the number of places, applications, interviews and offers made. This data was collected at a global level (and therefore at some institutions may have included more than one programme). There are missing data in the early years when this was not collected systematically. However, the data reveal some interesting trends:

- Between 2010 and 2018, the number of places available at UKCAT Consortium Universities increased from 5208 (Universities *n* = 25) to 6226 (*n* = 26) (+ 20%).
- There were fluctuations in reported applications (and changes in Consortium membership) during that period but the increase in applications between 2010 and 2018 was only 1367 (+ 2.4%). In 2018 there were 57,543 applications reported to UKCAT medical programmes (this figure includes applicants making multiple applications).
- The ratio of applications to places over this period has fallen (from 10.8 to 9.2).
- The number of interviews over this period has increased significantly from 14,795 to 23,057 (+ 56%) (this will include applicants being interviewed by more than one University).
- The ratio of interviews to places has increased (from 2.8 to 3.7).
- The number of offers made over this period has increased from 8952 to 14,146 (+ 58%).
- The ratio of offers to places has increased (from 1.7 to 2.3).

## Discussion

The UKCAT now represents a significant feature in selection to medicine in the UK, used by 30 out of 38 schools and in most contributes in a substantial way to selection outcomes. In 2018 a record 27,469 candidates took the test.

In the first years of testing, an unfamiliarity with the test led to an apparent reluctance on the part of schools to shift appreciably away from tried and tested selection methods. As a result, a large group of schools either made no use of the UKCAT or used the test in a light touch way, mainly to discriminate between applicants at a borderline for interview or offer.

Over time there has been a significant shift to use the test with greater emphasis. In 2018 23 out of 26 schools used the Threshold or Factor Method to select applicants for interview (2008, 16 out of 26). In 2018, the mean average weighting of the UKCAT to select applicants for interview was 39% (2008, 22%). By 2018, four schools used an actual UKCAT threshold to screen applicants at an early stage in selection processes. A further nine schools used a convenience threshold score to select applicants for interview. Whilst the threshold scores being used by schools increased a little over time, the average threshold scores remained around the mean average of the test. In 2018, 18 universities used the test at more than one stage in their selection processes.

The mean average weighting for personal statements and references has declined over this same period to 36% (2008, 58%) with only three Universities still using this criterion at this stage (2008, *n* = 9). It is of further interest to note that in 2008 16 schools either weighted or scored personal statements as part of their selection processes. In 2018, this figure was only three.

The mean average weighting for the academic record to select for interview has increased to 51% (2008, 39%).

### Drivers for change

Since 2006, there has been a shift away from the use of personal statements and references in selection. At the same time, Universities moved to greater reliance on a combination of academic achievement and potential, aptitude tests and multiple mini interviews. Whilst some of the shift has been a pragmatic one, this has been informed and influenced by some external drivers based on emerging evidence.

Many schools had for some time wanted to move away from a reliance on personal statements given the evidence that both these and references have limited predictive validity [9–11]. At the same time concern increased regarding the authenticity of personal statements given the growing coaching industry around medical student selection and the variation in advisor support for applicants [12].

Whilst the relationship is weak there have been papers published showing a significant positive relationship between the UKCAT and performance in medical schools [10, 11, 13–19] including evidence that the test has incremental validity over and above academic attainment [20].

Over this period of time a number of national reports provided additional guidance to schools looking to use best practice to inform their selection methods.

The Selection for Excellence Report [21] concluded that whilst insufficient evidence existed at the time to create a national framework for selection, there was enough evidence to advise schools to move towards processes combining academic attainment with performance in aptitude tests and MMIs. By 2018, all schools in the UK were using an admission test as part of their selection processes and as reported above, most used MMIs.

A systematic review of selection methods in medicine, building upon work commissioned by the Medical Schools Council, provided additional guidance for schools reviewing selection methods [22]. The review looked at eight different selection methods including aptitude tests, academic records, personal statements, situational judgement tests (SJTs) and interviews. The relative strengths of selection methods were discussed using four evaluation criteria: effectiveness (reliability and validity); procedural issues; acceptability, and cost-effectiveness. The authors concluded that academic records, MMIs, aptitude tests, SJTs and selection centres were more effective and generally fairer than traditional interviews, references and personal statements. However, the paper also reported ongoing challenges to the use of aptitude tests in selection, highlighting mixed evidence regarding predictive validity and fairness (noting some groups performed better in such tests), and the potential impact of coaching on performance.

On a more practical level, the UKCAT has provided schools with a simple tool for use in selection. Medical student selection is a time consuming and costly activity and Schools may have been attracted by the use of the test to narrow down applications requiring closer scrutiny. The UKCAT offers a simple, objective tool to discriminate between applicants. Whether used to reduce the number of UCAS forms to be reviewed, replacing the review of UCAS forms or used as a primary method of selection for interview, this undoubtedly streamlines and speeds up processing of applications.

### Widening access

There is limited and conflicting evidence regarding the effect use of the UKCAT might have on widening access to medicine. Tiffin concluded that strong use of the UKCAT might lead to more equitable distribution of offers across some under-represented groups [23] and that the UKCAT might be less sensitive to school type than A-levels [24]. However, a longitudinal review of the impact of the UKCAT could find no evidence of it reducing disadvantage [7]. Indeed, there would appear to be little evidence that changes to selection processes impact significantly on the demographic of applicants admitted to medical school [25]. More recently however, evidence that the UKCAT continues to predict undergraduate performance throughout medical schools (unlike school leaving qualifications) [26] may lead schools to consider greater use of the UKCAT as a contextual measure for applicants from low performing schools.

### Strengths and limitations

This comprehensive summary of entry requirements to UKCAT Consortium Medical Schools is a unique and valuable dataset. The completion rate is excellent but it is limited to those schools that use the UKCAT, which has shifted over time. Unfortunately, there is no information for schools that use or have used alternative admission tests or no admission test. The study focusses generally on UK applicants to the main programme offered by each medical school which in most cases will be an undergraduate 5 year course. There will be further variation in requirements for some subgroups of applicants (widening access, international) and other programmes (access, graduate entry).

The information reported regarding applicant numbers provides an interesting snapshot of fluctuations over time but ought to be treated with caution. This data was not always reported consistently and may have been interpreted differently by different schools.

### Implications for the future

Due to missing data (only UKCAT Universities are included and data was not collected systematically in the early years of UKCAT), applicant numbers reported above ought to be treated with caution. They do however reveal some interesting trends. Whilst the number of places available at medical schools continues to rise, it would appear that this is not necessarily matched by numbers of applicants. Universities appear to be interviewing significantly more applicants. It is perhaps the case that the increased places and the convergence of core selection criteria towards a focus on more objective measures (academic scores and UKCAT) and away from more subjective measures (personal statements) is impacting on conversion rates. That is, a reliance on these criteria results in more applicants being invited to multiple interviews than observed previously. Whilst a move away from a significant use of personal statements in selection is to be welcomed, this was one area where diversity in selection methods existed, with schools assessing forms in different ways. Whether Universities will wish to act around declining conversion rates (the proportion of applicants who accept an offer from an individual institution) remains to be seen.

Table 2 not only summarises use of the UKCAT in selection but illustrates the potential complexity which applicants are faced with when making their UCAS choices. It remains unclear what impact on outcomes this diversity in selection processes has but it is possible that the impact is fairly marginal (at a population level) and perhaps the sector ought to reflect on whether simpler approaches might create greater transparency for applicants. One advantage of the UKCAT is that applicants are aware of their scores prior to making their University choices. There ought to be sufficient information for applicants to avoid wasted applications. Greater exploration of features influencing applicant decision making such as that being undertaken in the UK Medical Applicant Cohort Study (UKMACS) [27] may influence future decisions regarding selection processes.

The creation of new medical schools, expansion of student numbers and a continued focus on widening access inevitably requires schools to continue to review selection processes. Researchers seeking a better understanding of the impact of the test on applicant demographics and the predictive validity of the test would benefit from an understanding of how schools use the UKCAT and how that use has changed since UKCAT’s inception. The study will be of interest to researchers investigating broader issues in medical school selection.

This paper does not seek to explore the rationales behind medical school decisions as to the nature of their selection processes. The growing evidence base around selection perhaps allows medical schools to reflect further on how different selection criteria align with the aims of their curricula and institutional values.

This paper demonstrates the benefit of understanding in detail how selection processes operate using information not systematically collected elsewhere. This level of detail ought to allow for more nuanced analysis of the impact of processes on applicant demographics and outcomes. If the UK Medical Education Database [28] is to be used to analyse the impact of selection on outcomes in medical school and beyond, then a greater understanding of different selection processes will be necessary. There would be particular benefit from collecting information regarding admissions to widening access programmes (and use of contextual data) from schools in order to be able to properly evaluate the success of such initiatives.

There is a tentative but growing use of the UKCAT SJT in selection, with some schools keen to include a measure of something other than academic achievement or potential. Other schools are awaiting further evidence regarding the predictive validity of this test.

## Conclusions

The UKCAT is now firmly established as part of the admissions landscape for medicine in the UK. A greater focus on the evidence base around selection has led to the UKCAT largely replacing the use of personal statements and references at most Universities. The use of the test by schools has grown, diversified and strengthened since 2006. Given the size of the UKCAT Consortium, the fate of most medical applicants will be in at least part determined by their performance in the test.

## Supplementary information

**Additional file 1: Supplementary Document 1.** Survey of medical schools’ use of the UKCAT.

**Additional file 2: Supplementary Document 2.** Uses of the UKCAT to select applicants for interview over time.

**Additional file 3: Supplementary Document 3.** Threshold and Factor Methods over time.

## Data Availability

The data that support the findings of this study are available upon reasonable request from the corresponding author RG.

## Abbreviations

HEFCE: Higher Education Funding Council for England
MMI: Multiple mini interview
PS&Ref: Personal statement and reference
SJT: Situational Judgement Test
UCAS: Universities and Colleges Admission Service
UCAT: University Clinical Aptitude Test
UKCAT: United Kingdom Clinical Aptitude Test
UKMACS: United Kingdom Medical Applicant Cohort Study
UKMED: UK Medical Education Database
WP: Widening participation
