# Supplementary material for: UKCAT and medical student selection in the UK – what has changed since 2006?
Source: BMC Med Educ. 2020 Sep 5;20:292. doi: 10.1186/s12909-020-02214-1 (PMC7487558; doi:10.1186/s12909-020-02214-1)
Supplement: Supplementary file 1 — Additional file 1: Supplementary Document 1. Survey of medical schools’ use of the UKCAT. [file 12909_2020_2214_MOESM1_ESM.pdf]

**<u>Survey of Medical Schools' use of the UKCAT (2018)</u>**

| | |
|---|---|
| **Medical School** | |
| **Name of respondent** | |
| **Role of respondent** | |
| **Telephone number** | |
| **Email** | |
| **Date and time of interview** | |
| **Interview duration** | |
| **Draft document sent for checking** | |
| **Final document received** | |

| **Basic statistics** | **Response in 2017** | **Response in 2018** |
|---|---|---|
| Number of places (home) | | |
| Number of places (overseas) | | |
| Number of applications | | |
| Number of interviews | | |
| Number of offers | | |
| Standard offer | | |

| | **Question** | **Response in 2017** | **Response in 2018** |
|---|---|---|---|
| 1 | Please describe your selection process this year. It may be useful to use the following headings: | | |
| | Receipt of form | | |
| | Screening academic credentials | | |
| | Reading PS and Ref | | |
| | Scoring | | |
| | Invitation for interview | | |
| | Format of interview | | |
| | Outcome of interview | | |
| | Recommendation for offer | | |
| | Making of offer | | |
| | Offer level | | |

| | Question | Response in 2017 | Response in 2018 |
|---|---|---|---|
| 2 | How did you use the UKCAT result in your selection process? | | |
| 2a | Was this used in pre-selection? | | |
| | Was this used in selection for interview? | | |
| | Was this used after interview? | | |
| 2b | Did you use it for assessment or selection of any specific subgroups? (eg borderline, WP, mature or disabled). Please specify and describe. | | |
| 3 | Did you make any other changes to your selection process this year? | | |
| 4a | Do you think using the UKCAT has affected the profile of the candidates you have selected this year, compared with previous years? If so, how? | | |
| 4b | Do you have any evidence to support this? | | |
| 5a | Have you analysed the effect of using the UKCAT on the profile of your selected candidates? If so, how? | | |
| 5b | Do you intend to analyse effect of using the UKCAT on the profile of your selected candidates? If so, how? | | |
| 6 | Are you anticipating any significant changes to your admission processes next year including the use of the UKCAT?<br><br>Please comment on whether you will be using the SJT results within your admission processes this year. | | |
