# Supplementary material for: UKCAT and medical student selection in the UK – what has changed since 2006?
Source: BMC Med Educ. 2020 Sep 5;20:292. doi: 10.1186/s12909-020-02214-1 (PMC7487558; doi:10.1186/s12909-020-02214-1)
Supplement: Supplementary file 2 — Additional file 2: Supplementary Document 2. Uses of the UKCAT to select applicants for interview over time. [file 12909_2020_2214_MOESM2_ESM.pdf]

ADDITIONAL FILE 2: Uses of the UKCAT to select applicants for interview over time

Different uses of the UKCAT to select applicants for interview over time

	Number of Medical Schools											
Year	Did not use	Borderline	Factor	Factor (inc SJT)	Factor and SJT Threshold	Threshold (actual and convenience)	Threshold (actual)	Threshold (actual) and factor	Threshold (actual) and SJT	Threshold (convenience)	Threshold (convenience) and SJT	Trade Off
**2007**	12	4	5				1			1		
**2008**	6	4	8				3			4		1
**2009**	6	3	8				1			7		1
**2010**	6	2	7					1		8		1
**2011**	6	1	7				1	1		8		1
**2012**	4	1	8				3	1		7		1
**2013**	3	1	9				2	2		8		
**2014**	1	2	7		2	1	3	2		7		
**2015**	1	1	7			1	3	3		7		
**2016**	2		7	1	1		3	2		8	1	
**2017**	2		8	1	1	1	1	2	1	7	1	
**2018**	3		7	1	2	1	1	2	1	7	1	

Total
23
26
26
25
25
25
25
25
23
25
25
26
