# Supplementary material for: UKCAT and medical student selection in the UK – what has changed since 2006?
Source: BMC Med Educ. 2020 Sep 5;20:292. doi: 10.1186/s12909-020-02214-1 (PMC7487558; doi:10.1186/s12909-020-02214-1)
Supplement: Supplementary file 3 — Additional file 3: Supplementary Document 3. Threshold and Factor Methods over time. [file 12909_2020_2214_MOESM3_ESM.pdf]

ADDITIONAL FILE 3: Threshold and Factor Methods over time

1 Mean Average Threshold Scores and UKCAT Scores over time

	Actual Threshold		Convenience Threshold		UKCAT (All Candidates)
Year	Average Score	Number of Schools	Average Score	Number of Schools	Average Score
2007	2350	1	2790	1	2375
2008	2403	4	2115	3	2401
2009	2600	1	2491	7	2430
2010	2250	2	2599	8	2505
2011	2319	4	2659	7	2489
2012	2397	6	2625	6	2476
2013	2511	6	2614	7	2516
2014	2591	6	2805	7	2643
2015	2468	6	2467	8	2505
2016	2369	5	2619	9	2531
2017	2449	4	2586	9	2523
2018	2463	4	2544	9	2540

2 Mean Average Weightings of Factors used in selection for interview over time

	UKCAT Weighting		Academic		Personal Statement & Reference		SJT	
Year	Number of Schools	Average Weighting %	Number of Schools	Average Weighting %	Number of Schools	Average Weighting %	Number of Schools	Average Weighting %
2007	4	26.11	9	29.38	9	58.02		
2008	7	22.48	9	38.56	9	58.04		
2009	7	20.88	10	35.03	9	55.74		
2010	7	20.88	9	36.43	8	51.03		
2011	7	23.47	9	40.58	8	45.70		
2012	8	31.05	10	44.35	7	43.39		
2013	10	37.58	10	44.85	5	33.23		
2014	10	40.50	9	53.23	4	31.46		
2015	9	43.70	9	52.81	1	23.53		
2016	10	42.40	10	53.72	1	22.54	2	9.61
2017	11	37.89	11	50.14	2	47.90	2	9.92
2018	11	39.39	11	50.81	2	36.11	2	10.28

3 Mean Average Weightings of Factors used in selection for offer over time

	Interview Score		UKCAT		Academic		Personal Statement & Reference		SJT	
Year	Number of Schools	Average Weighting %	Number of Schools	Average Weighting %	Number of Schools	Average Weighting %	Number of Schools	Average Weighting %	Number of Schools	Average Weighting %
2007	23	91.12	2	8.36	3	44.33	3	33.17		
2008	24	89.82	3	8.91	3	40.64	3	33.17		
2009	24	89.82	3	8.91	3	40.64	3	31.31		
2010	23	91.83	4	9.43	2	49.92	2	29.87		
2011	23	91.38	4	20.78	2	46.62	2	31.82		
2012	23	89.44	4	20.55	2	46.62	3	36.37		
2013	23	89.64	4	24.53	2	46.62	3	29.55		
2014	24	90.18	5	23.93	2	50.00	3	15.00	1	15.00
2015	22	95.45	2	35.00	1	50.00	1	15.00	1	15.00
2016	24	94.14	3	28.10	1	50.00	1	15.00	3	13.75
2017	24	93.68	3	28.10	1	50.00	1	15.00	4	13.09
2018	25	90.93	4	27.32	2	50.00	1	15.00	4	13.09
